# Short-term and long-term outcomes after robotic versus open hepatectomy in patients with large hepatocellular carcinoma: a multicenter study

**DOI:** 10.1097/JS9.0000000000000873

**Published:** 2023-11-16

**Authors:** Xiu-Ping Zhang, Nan Jiang, Lin Zhu, Zhao-Yi Lin, Wei-Xing Guo, Xiong Chen, Yun-Tao Ma, Fan Zhang, Yu-Fu Tang, Zi-Li Chen, Mao-Lin Yan, Zhi-Ming Zhao, Cheng-Gang Li, Wan Yee Lau, Shu-Qun Cheng, Ming-Gen Hu, Rong Liu

**Affiliations:** a Faculty of Hepato-Biliary-Pancreatic Surgery, Chinese People’s Liberation Army (PLA) General Hospital; Institute of Hepatobiliary Surgery of Chinese PLA; Key Laboratory of Digital Hepatobiliary Surgery, PLA, Beijing; b The First Clinical Medical School, Lanzhou University; c Department of General Surgery, Gansu Provincial Hospital, Lanzhou, Gansu; d Department of Hepatobiliary Surgery, People’s Hospital of Xinjiang Uygur Autonomous Region, Urumqi, Xinjiang; e Department of Hepatic Surgery VI, Eastern Hepatobiliary Surgery Hospital, Second Military Medical University, Shanghai; f Department of Hepatobiliary Surgery, Affiliated Hospital of Binzhou Medical College, Shandong, China Department of Hepato-Biliary-Pancreatic Surgery; g Department of Hepato-Biliary-Pancreatic Surgery, Fujian Provincial Hospital, Fujian; h Department of Hepatobiliary Surgery, Affiliated Hospital of Guizhou Medical University, Guizhou; i Department of Hepatobiliary Surgery, Northern Theatre General Hospital, Liaoning; j Faculty of Medicine, the Chinese University of Hong Kong, Shatin, Hong Kong, SAR, People’s Republic of China

**Keywords:** large hepatocellular carcinoma, open hepatectomy, outcomes, propensity score matching, robotic hepatectomy

## Abstract

**Background:**

Robotic hepatectomy (RH) is currently widely accepted and it is associated with some benefits when compared to open hepatectomy (OH). However, whether such benefits can still be achieved for patients with large hepatocellular carcinoma (HCC) remain unclear. This study aimed to evaluate the short-term and long-term outcomes of patients undergoing RH or OH.

**Methods:**

Perioperative and survival data from patients with large HCC who underwent RH or OH between January 2010 and December 2020 were collected from eight centres. Propensity score matching (PSM) was performed to minimise potential biases.

**Results:**

Using predefined inclusion criteria, 797 patients who underwent OH and 309 patients who underwent RH were enroled in this study. After PSM, 280 patients in the robotic group had shorter operative time (median 181 vs. 201 min, *P*<0.001), lower estimated blood loss (median 200 vs. 400 ml, *P*<0.001), and shorter postoperative length of stay (median 6 vs. 9 days, *P*<0.001) than 465 patients in the open group. There were no significant differences between the two groups in overall survival and recurrence-free survival. Cox analysis showed AFP greater than 400 ng/ml, tumour size greater than 10 cm, and microvascular invasion were independent risk factors for overall survival and recurrence-free survival. After PSM, subgroup analysis showed that patients with a huge HCC (diameter >10 cm) who underwent RH had significantly lower estimated blood loss (median 200.0 vs. 500.0 min, *P*<0.001), and shorter length of stay (median 7 vs. 10 days, *P*<0.001) than those who underwent OH.

**Conclusion:**

Safety and feasibility of RH and OH for patients with large HCC were comparable. RH resulted in similar long-term survival outcomes as OH.

## Introduction

Highlights

- This multicenter study aimed to evaluate the short-term and long-term outcomes of patients with large hepatocellular carcinoma undergoing robotic hepatectomy (RH) or open hepatectomy (OH).
- Safety and feasibility of RH and OH for these patients were comparable.
- RH resulted in similar long-term survival outcomes as OH.

Hepatocellular carcinoma (HCC) is a very common cancer in the world with a high rate of cancer-related mortality^1^. Hepatectomy is the main first-line treatment for HCC, which can offer patients the chance of long-term survival^2,3^. With advances in surgical techniques and knowledge related to liver surgery, tumour size is no longer considered to be a contraindication to hepatectomy. Many experienced centres have reported on hepatectomy for HCC larger than 5 cm, or even 10 cm^4,5^. Previous studies have classified liver tumours larger than 5 cm and 10 cm as ‘large’ or ‘huge’ HCC, respectively^6,7^.

The high recurrence rate of HCC after hepatectomy results in unsatisfactory long-term survival outcomes of these patients^8^. Tumour size is recognised to be a very important prognostic factor for survival and recurrence. It forms the basis of tumour staging systems, and at least to a certain extent, guide the treatment of HCC^6,9^. Furthermore, tumour size is also a potential risk factor affecting positive resection margins in hepatectomy^10^. Results for tumours that are larger than 5 cm, studies reported contradictory results. While some studies suggested that tumour size exceeding 5 cm to be a poor prognostic factor, others reported that there were no significant impact on long-term survival for patients with tumours larger than 5 cm^6,11–13^.

Minimally invasive hepatectomy is now considered to be a safe and effective treatment for liver tumours in experienced hands^14^. In addition, it results in more rapid postoperative recovery, allowing patients to receive adjuvant oncological treatment without delay^15^. Rapid development of robotic hepatectomy (RH) has significantly changed the landscape of liver resection^16^. RH, including robotic donor hepatectomy, robotic major hepatectomy, and robotic minor hepatectomy, is increasingly used in many specialized centres^17–21^. The robotic system can provide better flexibility, ergonomics, and three-dimensional magnified views of the surgical site^22,23^, contributing to lower conversion rates and shorter length of stay (LOS) in hospital^24^. Our centre is a high-volume centre with extensive experience in both RH and OH^20,25–27^. Tumour size is known to be a factor impacting the difficulty of hepatectomy, especially in minimally invasive liver resection^28^, Furthermore, there is a lack of data on the short-term and long-term outcomes of RH in patients with large tumours compared with open hepatectomy (OH).

This multicenter study aimed to compare the short-term and long-term outcomes of RH and OH in patients with large HCCs in the different clinical subgroups to provide data to facilitate overall management of patients with large HCC.

## Methods

### Patients

This retrospective multicenter study was carried out on consecutive HCC patients with large HCCs who underwent RH or OH between January 2010 and December 2020 in eight centres. The inclusion criteria were patients: (1) with a resectable tumour 5 cm or greater which was histopathologically confirmed as HCC; (2) aged over 18 years; and (3) with no anaesthesia or surgical contraindications. The exclusion criteria were patients with: (1) other types of malignant tumours; (2) distant metastases; (3) unavailable perioperative data; (4) combined extrahepatic resection; and (4) intraoperative open conversion. The study was approved by all the Hospital’s Institutional Review Boards. The ethics committee waived the informed consent requirement due to the anonymity of patient identities. This retrospective cohort study was registered with ResearchRegistry.com. The work was reported in accordance with the strengthening the reporting of cohort, cross-sectional and case–control studies in surgery (STROCSS) criteria^29^ (Supplemental Digital Content 1, http://links.lww.com/JS9/B314).

### Perioperative data

Guidelines on the diagnosis and treatment plan of HCC were designed by a multidisciplinary team and these were followed by each of the multiple centres. The data was collected from an electronic database and the data were subsequently analysed retrospectively. The data included perioperative baseline characteristics, pathology, and surgical outcomes. A tumour size of 5 cm or larger was considered to be a large HCC while a tumour size exceeding 10 cm was defined as a huge HCC^30–32^. Microvascular invasion was defined as presence of tumour emboli within the central vein, portal vein, or large capsular vessels or involvement of segmental or sectoral branches of portal or hepatic veins^33,34^. Morbidity grade was estimated using the Clavien–Dindo classification, and major complications were defined as a Clavien–Dindo grade ≥ 3^35^. We used the BCLC staging system, which was widely used and had an updated version in 2022^36^. The estimated blood loss was calculated based on the difference between the suction canister fluids and the abdominal irrigation fluids, plus the difference in weight between operative and dry gauze^37^.

### Surgical procedures and follow-up

All RH procedures were performed by surgeons who had completed more than 30 robotic liver resections and had surpassed the associated learning curve^38^. Robotic surgical techniques, including patient positioning and robotic settings, have been described in our previous articles^25,39,40^. The follow-up strategy, including follow-up interval and content, was the same as in our previously reported study^8^. Overall survival (OS) was defined as the time interval from surgery to death or the last follow-up, and recurrence-free survival (RFS) was defined as the time from resection to the date of first diagnosis or the last follow-up for tumour recurrence. This study was censored on 30 June 2021.

### Statistical analysis

Categorical variables were presented as numbers and percentages. Continuous variables were manifested as medians and interquartile ranges. Baseline, operative, and postoperative data comparison between the robotic and open groups were performed using the Mann–Whitney *U* test for continuous variables and the χ^2^test or Fisher’s exact test for categorical variables. PSM was used to reduce selection bias between groups and subgroup analyses were performed based on tumour sizes to study the impact of surgical methods on selected patients. For PSM, the caliper width was set to a propensity score of 0.1 SD, and patients were matched to controls in a 1:2 ratio. A standardized mean differences dot plot was used to display the results of balanced tests. Univariable and multivariable Cox proportional hazards models were used to analyse potential prognostic variables. Hazard ratios (HRs) and 95% CI were reported to measure the effects of potential prognostic variables. Survival analysis was calculated using Kaplan–Meier (K–M) analysis and compared using the log-rank test. A *P*<0.05 was considered statistically significant. All statistical analyses were performed using SPSS software (version 22.0) and R software (version 4.1.1).

## Results

### Patient characteristics

Of 797 patients who were included in the OH group and 309 patients who were included in the RH group; the huge HCC subgroup consisted of 347 patients, of which 299 underwent OH and 48 underwent RH. The patient characteristics in the unmatched cohorts differed significantly in AFP (*P*<0.001), varices rates (11 vs. 4%, *P*<0.001), tumour size (*P*<0.001) and MVI positive rates (56 vs. 42%, *P*<0.001). PSM was carried out to reduce selection bias. The matched results were not in an absolute ratio of 1:2 because there were no matched objects in some cases. A standardized dot plot of the mean differences for PSM is shown in Supplementary Figure 1 (Supplemental Digital Content 2, http://links.lww.com/JS9/B315). After PSM, the open and robotic groups featured 465 patients and 280 patients, respectively. There were no significant differences in the baseline characteristics between the two groups. The baseline characteristics of HCC patients with large tumours in the robotic and open groups before and after PSM are shown in Table 1.

**Table 1:** Baseline characteristics of HCC patients with large tumours in the robotic and open groups before and after PSM.

	Before PSM			After PSM^a^		
Variable	Open group (*n*=797)	Robotic group (*n*=309)	*P*	Open group (*n*=465)	Robotic group (*n*=280)	*P*
Age, years						
≤60	605 (76%)	172 (56%)	<0.001	316 (68%)	172 (61%)	0.069
>60	192 (24%)	137 (44%)		149 (32%)	108 (39%)	
Sex						
Female	127 (16%)	58 (19%)	0.257	80 (17%)	48 (17%)	0.983
Male	670 (84%)	251 (81%)		385 (83%)	232 (83%)	
BMI, kg/m^2^	24.19 (22.66–26.4)	24.22 (22.16–26.08)	0.418	24.21 (22.6–26.17)	24.26 (22.45–25.93)	0.883
ASA grade						
≤Ⅱ	738 (93%)	280 (91%)	0.274	429 (92%)	256 (91%)	0.687
>Ⅱ	59 (7%)	29 (9%)		36 (8%)	24 (9%)	
Viral hepatitis						
No	61 (8%)	57 (18%)	<0.001	48 (10%)	31 (11%)	0.748
Yes	736 (92%)	252 (82%)		417 (90%)	249 (89%)	
Cirrhosis						
No	287 (36%)	112 (36%)	0.942	172 (37%)	94 (34%)	0.346
Yes	510 (64%)	197 (64%)		293 (63%)	186 (66%)	
ALB, g/l						
<35	80 (10%)	32 (10%)	0.875	46 (10%)	32 (11%)	0.507
≥35	717 (90%)	277 (90%)		419 (90%)	248 (89%)	
TBIL, mol/l						
≤17	602 (76%)	243 (79%)	0.275	361 (78%)	215 (77%)	0.789
>17	195 (24%)	66 (21%)		104 (22%)	65 (23%)	
INR	1.08 (1.01–1.16)	1.07 (1.02–1.13)	0.610	1.07 (1.01–1.15)	1.07 (1.02–1.14)	0.361
AFP, ng/ml						
≤400	441 (55%)	208 (67%)	<0.001	295 (63%)	182 (65%)	0.668
>400	356 (45%)	101 (33%)		170 (37%)	98 (35%)	
BCLC stage						
A	719 (90%)	274 (89%)	0.448	407 (88%)	247 (88%)	0.781
B	78 (10%)	35 (11%)		58 (12%)	33 (12%)	
Varices						
No	711 (89%)	298 (96%)	<0.001	444 (95%)	269 (96%)	0.702
Yes	86 (11%)	11 (4%)		21 (5%)	11 (4%)	
Lesion size, cm						
≤ 10	498 (62%)	261 (84%)	<0.001	376 (81%)	232 (83%)	0.496
>10	299 (38%)	48 (16%)		89 (19%)	48 (17%)	
No. of tumours						
Solitary	719 (90%)	274 (89%)	0.448	407 (88%)	247 (88%)	0.781
Multiple	78 (10%)	35 (11%)		58 (12%)	33 (12%)	
MVI						
Absent	349 (44%)	178 (58%)	<0.001	228 (49%)	156 (56%)	0.077
Present	448 (56%)	131 (42%)		237 (51%)	124 (44%)	

Data are presented as *n* (%) or median (interquartile range).

a because some cases could not simultaneously find effective matching objects, the matching result was not an absolute 1:2.

AFP, α-fetoprotein; ALB, albumin; ASA, American Society of Anesthesiologists; BCLC stage, Barcelona Clinic Liver Cancer stage; HCC, hepatocellular carcinoma; INR, international normalised ratio; MVI, microvascular invasion; PSM, propensity score matching; TBIL, total bilirubin.

### Comparison of short-term outcomes between the RH and OH groups

The surgical outcomes of HCC patients with large tumours in the robotic and open groups before and after PSM are shown in Table 2. After PSM, there was no significant difference in total clamping time (median 21 vs. 26 min, *P*=0.380). However, the robotic group had lower operative time (median 181 vs. 201 min, *P*<0.001), lower estimated blood loss (median 200 vs. 400 ml, *P*<0.001), decreased postoperative LOS (median 6 vs. 9 days, *P*<0.001), and a lower major complication rate (2 vs. 6%, *P*=0.014). Details on the short-term outcomes before and after PSM are shown in Table 2.

**Table 2:** Surgical outcomes of HCC patients with large tumours in the robotic and open groups before and after PSM.

	Before PSM				After PSM			
Variable	Open group (*n*=797)	Robotic group (*n*=309)		*P*	Open group (*n*=465)	Robotic group (*n*=280)		*P*
Pringle manoeuvre								
No	172 (22%)	96 (31%)		0.001	94 (20%)	85 (30%)		<0.001
Yes	625 (78%)	213 (69%)			371 (80%)	195 (70%)		
Total clamping time, min	24 (9–41)	25 (0–41)		0.687	21 (11–37)	26 (0–42)		0.380
Operative time, min	210 (180–275)	180 (130–230)		<0.001	201 (180–245)	181 (130–230)		<0.001
Estimated blood loss, ml	400 (200–800)	100 (100–300)		<0.001	400 (200–800)	200 (100–300)		<0.001
Postoperative LOS, days	9 (8–13)	6 (5–8)		<0.001	9 (8–12)	6 (5–8)		<0.001
Clavien–Dindo grade >II								
No	747 (94%)	302 (98%)		0.007	437 (94%)	274 (98%)		0.014
Yes	50 (6%)	7 (2%)			28 (6%)	6 (2%)		

Data are presented as *n* (%) or median (interquartile range).

HCC, hepatocellular carcinoma; LOS, length of stay; PSM, propensity score matching.

### Comparison of long-term outcomes between the RH and OH groups

**Figure 1:**
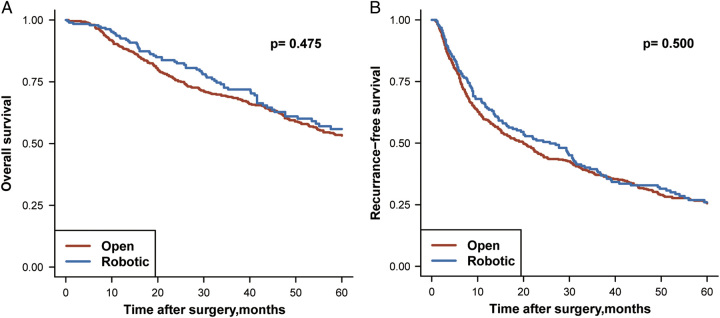
Kaplan–Meier curves estimating OS and RFS of HCC patients after PSM.

Patients in the robotic group and the open group had similar OS (*P*=0.475) and RFS (*P*=0.500) after PSM. The median OS was 64.4 months in the open group and 68.9 months in the robotic group. The 1-year, 3-year, and 5-year OS rates were 89.2, 68.9, and 53.2% in the open group, and 92.5, 71.9, and 55.9% in the robotic group, respectively. The median RFS was 20.0 months in the open group and 25.7 months in the robotic group. The 1-year, 3-year, and 5-year RFS rates were 59.4, 37.1, and 25.6% in the open group, and 64.7, 38.8, and 26.0% in the robotic group, respectively. Detailed comparison of long-term outcomes between the robotic and open groups are shown in Figure 1 and Supplementary Table 1 (Supplemental Digital Content 2, http://links.lww.com/JS9/B315).

### Univariable and multivariable Cox regression analyses on survival outcomes in all HCC patients after hepatectomy

**Figure 2:**
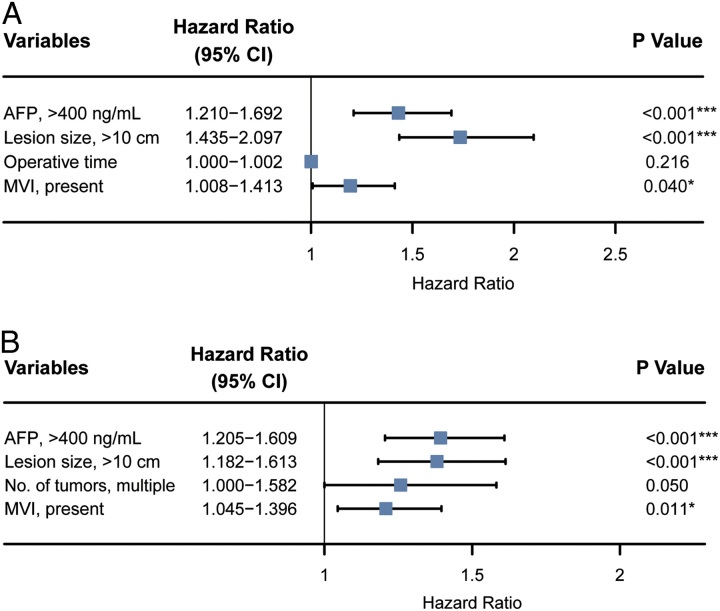
Forest plots of multivariable analysis for OS and RFS for patients with large tumours. A. forest plot of multivariable analysis for OS; B. forest plot of multivariable analysis for RFS.

Univariable and multivariable Cox regression analysis for OS of all the HCC patients in the study are shown in Table 3. AFP greater than 400 ng/ml (HR=1.431, *P*<0.001), HCC size greater than 10 cm (HR=1.735, *P*<0.001), and presence of MVI (HR=1.194, *P*=0.040) were identified as independent risk factors of OS. Univariable and multivariable Cox regression analyses of RFS for HCC patients with large tumours are shown in Supplementary Table 2 (Supplemental Digital Content 2, http://links.lww.com/JS9/B315). AFP greater than 400 ng/ml (HR=1.393, *P*<0.001), HCC size greater than 10 cm (HR=1.381, *P*<0.001), and presence of MVI (HR=1.208, *P*=0.011) were identified as independent risk factors of RFS in all HCC patients. The multivariable Cox regression analysis results are shown in Figure 2.

**Table 3:** Univariable and multivariable cox proportional hazards analyses for OS.

	Univariable analysis		Multivariable analysis	
Characteristics	HR (95% CI)	*P*	HR (95% CI)	*P*
Age, years, >60 vs ≤60	0.852 (0.707–1.027)	0.092		
Sex, male versus female	0.905 (0.724–1.131)	0.382		
BMI, kg/m^2^	0.982 (0.954–1.011)	0.219		
ASA grade, III vs ≤II	0.887 (0.651–1.207)	0.446		
Viral hepatitis, yes versus no	1.202 (0.874–1.652)	0.257		
ALB, g/l, ≥35 versus <35	0.850 (0.652–1.109)	0.231		
TBIL, mol/l, >17 versus ≤17	1.071 (0.884–1.297)	0.484		
AFP, ng/ml, >400 versus ≤400	1.512 (1.280–1.786)	<0.001	1.431 (1.210–1.692)	**<0.001**
Lesion size, cm, >10 versus ≤10	1.851 (1.542–2.220)	<0.001	1.735 (1.435–2.097)	**<0.001**
MVI, present versus absent	1.216 (1.028–1.439)	0.023	1.194 (1.008–1.413)	**0.040**
Operative time, min	1.001 (1.000–1.003)	0.005	1.001 (1.000–1.002)	0.216
Clavien–Dindo grade >II, yes versus no	1.366 (0.982–1.900)	0.064		

Bold text hinted that these variables were statistically significant.

AFP, α-fetoprotein; ALB, albumin; ASA, American Society of Anesthesiologists; HR, Hazard Ratio; MVI, Microvascular invasion; OS, overall survival; TBIL, total bilirubin.

### Subgroup analysis of patients with huge HCC (diameter >10 cm)

**Figure 3:**
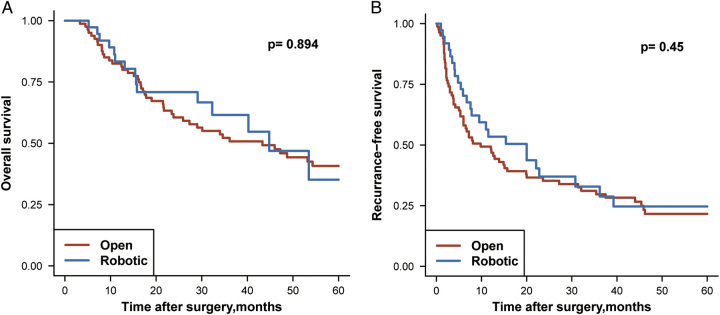
Kaplan–Meier curves estimating OS and RFS for HCC patients after PSM in the huge HCC subgroup.

The baseline characteristics of patients in the huge subgroup are shown in Supplementary Table 3 (Supplemental Digital Content 2, http://links.lww.com/JS9/B315). There were 347 patients in this subgroup, with 299 patients in the open group, and 48 patients in the robotic group. After 1:2 PSM, 83 patients in the open group and 47 patients in the robotic group were matched. For patients in the huge HCC subgroup, the robotic group had shorter operative time (median 220 vs. 250 min, *P*=0.005), lower estimated blood loss (median 200 vs. 500 ml, *P*<0.001) and shorter postoperative LOS (median 7 vs. 10 days, *P*<0.001) when compared with the open group. However, unlike the short-term prognosis of the large HCC subgroup, there was no significant difference in the major complication rate (*P*=0.477). The details are shown in Table 4. On subgroup analysis, OS and RFS were similar for patients in the huge HCC subgroup who underwent the two different surgical approaches. K–M plots and details of the long-term outcomes in the subgroup analysis are shown in Figure 3 and Supplementary Table 4 (Supplemental Digital Content 2, http://links.lww.com/JS9/B315).

**Table 4:** Surgical outcomes of HCC patients with huge tumours (>10 cm) in the robotic and open groups before and after PSM.

	Before PSM			After PSM^a^		
Variable	Open group (*n*=299)	Robotic group (*n*=48)	*P*	Open group (*n*=83)	Robotic group (*n*=47)	*P*
Pringle manoeuvre						
No	55 (18%)	14 (29%)	0.083	14 (17%)	13 (28%)	0.145
Yes	244 (82%)	34 (71%)		69 (83%)	34 (72%)	
Total clamping time, min	34 (13.5–57)	30 (0–48)	0.376	31 (16–58)	30 (0–48)	0.488
Operative time, min	245 (190–309.5)	218 (170–270)	0.006	250 (200–318)	220 (173–270)	0.005
Estimated blood loss, ml	500 (300–1000)	200 (100–325)	<0.001	500 (300–1000)	200 (100–350)	<0.001
Postoperative LOS, days	9 (8–14)	7 (6–9)	<0.001	10 (8–13)	7 (6–9)	<0.001
Clavien–Dindo grade >II						
No	278 (93%)	48 (100%)	0.117	80 (96%)	47 (100%)	0.477
Yes	21 (7%)	0 (0%)		3 (4%)	0 (0%)	

Data are presented as *n* (%), and median (IQR).

a because some cases could not simultaneously find effective matching objects, the matching result was not an absolute 1:2.

HCC, hepatocellular carcinoma; IQR, interquartile range; LOS, length of stay; PSM, propensity score matching.

## Discussion

Hepatectomy remains the best option for treatment of large liver cancers^32^. With development of the robotic surgical systems and optimisation of surgical techniques, RH is becoming increasingly deployed for such surgeries. As a considerable proportion of HCC patients have large tumours^6,7^, and a large tumour size increases the difficulty of hepatectomy, minimally invasive hepatectomy faces unique technical challenges including distortion of normal anatomy, compression of vessels, increased number of tumour-supplying vessels, and presence of tumour invasion^7^. When compared with OH, previous studies have indicated that minimally invasive hepatectomy to be associated with lower estimated blood loss and shorter hospital stay, and with similar safety and efficiency for patients with large HCCs^2,4,28,41–43^. However, few studies have reported the short-term and long-term outcomes of RH in patients with large tumours. It is still unclear as to whether these patients would benefit from robotic surgery.

In our study, the short-term and long-term outcomes of RH was evaluated to compare with OH in patients with large HCCs. This retrospective, multicenter study was used to study the surgical outcomes of RH versus OH, and PSM was carried out to reduce selection bias. Since the prognosis of large HCCs after hepatectomy is worse than that of smaller HCCs^6^, subgroup analysis was used to evaluate whether the short-term or long-term results differ significantly between these two groups in relation to tumour size. Our study demonstrated that RH had better short-term outcomes, but with similar long-term outcomes when compared with OH. AFP greater than 400 ng/l, HCC size greater than 10 cm, and presence of MVI were found to be independent risk factors for OS and RFS. Subgroup analysis further demonstrated that RH showed better results for the RH group than the OH group in the huge HCC subgroup, implying that RH for these patients is worthy of consideration.

After PSM, the preoperative characteristics were similar in the two groups. The robotic group still showed significantly better short-term outcomes, including lower estimated blood loss and shorter postoperative LOS. These findings are consistent with the results reported in most previous studies. The decreased estimated blood loss may be related to the haemostatic effect of pneumoperitoneum and meticulous haemostasis under three-dimensional visualisation. Furthermore, based on a previous study, laparoscopic hepatectomy for large HCCs tended to have longer operative time than OH (297.5 vs. 205 min, *P*<0.001)^5^. Our study indicated that RH typically took less time to perform than laparotomic hepatectomy (181 vs. 201 min, *P*<0.001), suggesting that the robotic systems can offer some benefits and can enable surgeons to carry out hepatectomy more efficiently and with better outcomes.

Previous studies have identified tumour size and vascular invasion as independent predictors of survival for large HCCs^32,43^. Similar results were obtained in our present study. Furthermore, AFP levels, HCC size, and presence of MVI were shown to be independent risk factors for OS and RFS. Advanced HCCs with high AFP levels and presence of MVI have been shown to be associated with poor prognosis^44^.

Previous studies have shown a 5-year OS rate for large HCC to range from 32.3–77.9%^31,32,45,46^. Our study showed that patients with large HCCs had a high recurrence rate. Thus, these patients require regular postoperative surveillance to provide early appropriate treatments when recurrence is detected. In our study, there was no significant difference between the robotic and open groups, suggesting that RH to be a good treatment option for patients with large HCC. Our results were consistent with the results obtained in previous studies in that the surgical approach (RH vs. OH) did not significantly affect OS and RFS in patients with large HCCs^5^. On subgroup analysis, RH was better in estimated blood loss, perioperative LOS, and decreased operative time. These outcomes indicated that RH to be safe and feasible to be used as a treatment option for patients with large HCCs.

The guidelines in different areas have some differences, and one of the main differences between Western and Eastern guidelines is the criteria for surgical resection^47–49^. The European Association for the Study of the Liver (EASL) Clinical Practice Guidelines state that only patients with a single lesion less than 5 cm in maximum diameter, or a maximum of three lesions with a maximum diameter of 3 cm each, are eligible for surgical resection. However, some Asian guidelines, such as Korean, Japanese, and Chinese guidelines, allow hepatectomy for patients with larger or multiple lesions, as long as they have well-preserved hepatic function, no main portal trunk invasion, and no extrahepatic spread. The differences between Western and Eastern guidelines for hepatectomy of HCC reflect the differences in the prevalence of HCC, local clinical practice, insurance system, and other availability of resources^50^. Therefore, the differences between Western and Eastern clinical practices may limit the use of our results. According to the guidelines, the treatment options for HCC depend on several factors, such as the size, number, and location of the lesions, the stage of the cancer, the liver function, the general health, and patients’ preferences. The main aim of treatment is to achieve prolonged survival and improve quality of life. A recent study provides preliminary evidence for the safety and efficacy of combined therapy for huge HCC^51^. The treatment of large HCCs will involve multiple disciplines and become more personalised with the development of high-quality clinical studies.

This study has some limitations. First, this is a retrospective study with potential bias. Second, the majority of HCC patients in this study suffered from viral hepatitis B, the aetiological factor differs significantly from HCC patients in western countries. Therefore, our results need to be validated in western patient cohorts. Finally, the centres involved in this study are high-volume centres which may limit the use of our results to other centres.

In conclusion, this study demonstrated the feasibility and safety of RH in HCC patients with large tumours. RH showed comparable short-term and long-term outcomes when compared with OH.

## Ethics approval and consent to participate

This study was approved by the Ethics Committee of the PLA General Hospital and Chinese Alliance of Hepato-Biliary-Pancreatic Surgery (S2016-098-02). Written informed consent was obtained from all patients. This retrospective cohort study was registered with ResearchRegistry.com (Unique Identification Number: researchregistry 9295).

## Consent for publication

Consent for publication was obtained from all authors.

## Sources of funding

This work was supported by the China National Key R&D Program during the 14th Five-year Plan Period (Grant No.2022YFC2407403).

## Author contribution

R.L., W.Y.L., M.-G.H., S.-Q.C., and X.-P.Z.: conception and design; R.L., S.-Q.C., and M.-G.H.: financial support; N.J., L.Z., Z.-Y.L., W.-X.G., X.C., Y.-T.M., F.Z., Y.-F.T., Z.-L.C., M.-L.Y., Z.-M.Z.: provision of study materials or patients; X.-P.Z., N.J., L.Z., and Z.-Y.L.: collection and assembly of data; X.-P.Z., N.J., and L.Z.: data analysis and interpretation; X.-P.Z., W.Y.L.: manuscript writing. All authors contributed in final approval of manuscript.

## Conflicts of interest disclosure

No potential conflicts of interest were disclosed.

## Research registration unique identifying number (UIN)

1. Name of the registry: Short-term and Long-term Outcomes after Robotic versus Open Hepatectomy in Patients with Large Hepatocellular Carcinoma: A Multicenter Study.
2. Unique identifying number or registration ID: researchregistry9295.
3. Hyperlink to your specific registration (must be publicly accessible and will be checked): https://www.researchregistry.com/browse-theregistry#home/registrationdetails/64b8f83d7bb4300029c0970f/

## Guarantor

Prof. Rong Liu, M.D., Ph.D. Faculty of Hepato-Biliary-Pancreatic Surgery, Chinese People’s Liberation Army. (PLA) General Hospital. 28 Fuxing Road, Beijing 100853, People’s Republic of China. Tel: +86 10 66939377; fax: +86 10 66939377. E-mail: liurong301@126.com

## Data availability statement

The data used and/or analysed during the current study are available from the corresponding author on reasonable request.

## Supplementary Material

## References

[1] Siegel RL Miller KD Fuchs HE . Cancer statistics, 2021. CA Cancer J Clin 2021;71:7–33. 33433946 10.3322/caac.21654

[2] Troisi RI Berardi G Morise Z . Laparoscopic and open liver resection for hepatocellular carcinoma with Child-Pugh B cirrhosis: multicentre propensity score-matched study. Br J Surg 2021;108:196–204. 33711132 10.1093/bjs/znaa041

[3] Suzuki Y Naganuma A Hoshino T . Tolvaptan reduces the required amount of albumin infusion in patients with decompensated cirrhosis with uncontrolled ascites : a multicenter retrospective propensity score-matched cohort study. Acta Gastroenterol Belg 2021;84:57–63. 33639694 10.51821/84.1.357

[4] Kabir T Syn NL Guo Y . Laparoscopic liver resection for huge (>/=10 cm) hepatocellular carcinoma: a coarsened exact-matched single-surgeon study. Surg Oncol 2021;37:101569. 33839442 10.1016/j.suronc.2021.101569

[5] Peng Y Li B Xu H . Is the anterior approach suitable for laparoscopic right hemihepatectomy in patients with large HCC (5-10 cm)? A propensity score analysis. Surg Endosc 2022;36:6024–6034. 35182216 10.1007/s00464-022-09119-8

[6] Chang YJ Chung KP Chang YJ . Long-term survival of patients undergoing liver resection for very large hepatocellular carcinomas. Br J Surg 2016;103:1513–1520. 27550624 10.1002/bjs.10196

[7] Cheung TT Wang X Efanov M . Minimally invasive liver resection for huge (>/=10 cm) tumors: an international multicenter matched cohort study with regression discontinuity analyses. Hepatobiliary Surg Nutr 2021;10:587–597. 34760963 10.21037/hbsn-21-327 PMC8527431

[8] Zhang XP Xu S Hu MG . Short- and long-term outcomes after robotic and open liver resection for elderly patients with hepatocellular carcinoma: a propensity score-matched study. Surg Endosc 2022;36:8132–8143. 35534731 10.1007/s00464-022-09236-4

[9] Mazzaferro V Bhoori S Sposito C . Milan criteria in liver transplantation for hepatocellular carcinoma: an evidence-based analysis of 15 years of experience. Liver Transpl 2011;17(Suppl 2):S44–S57. 21695773 10.1002/lt.22365

[10] Benedetti Cacciaguerra A Gorgec B Cipriani F . Risk factors of positive resection margin in laparoscopic and open liver surgery for colorectal liver metastases: a new perspective in the perioperative assessment. A European Multicenter Study Ann Surg 2022;275:e213–e221. 10.1097/SLA.0000000000004077 32657916

[11] Liao W Zhang J Zhu Q . Preoperative neutrophil-to-lymphocyte ratio as a new prognostic marker in hepatocellular carcinoma after curative resection. Transl Oncol 2014;7:248–255. 24704092 10.1016/j.tranon.2014.02.011 PMC4101343

[12] Arnaoutakis DJ Mavros MN Shen F . Recurrence patterns and prognostic factors in patients with hepatocellular carcinoma in noncirrhotic liver: a multi-institutional analysis. Ann Surg Oncol 2014;21:147–154. 23959056 10.1245/s10434-013-3211-3 PMC4048028

[13] Lim C Mise Y Sakamoto Y . Above 5 cm, size does not matter anymore in patients with hepatocellular carcinoma. World J Surg 2014;38:2910–2918. 25099682 10.1007/s00268-014-2704-y

[14] Buell JF Cherqui D Geller DA . The international position on laparoscopic liver surgery: The Louisville Statement, 2008. Ann Surg 2009;250:825–830. 19916210 10.1097/sla.0b013e3181b3b2d8

[15] Kawai T Goumard C Jeune F . Laparoscopic liver resection for colorectal liver metastasis patients allows patients to start adjuvant chemotherapy without delay: a propensity score analysis. Surg Endosc 2018;32:3273–3281. 29340819 10.1007/s00464-018-6046-y

[16] Gotohda N Cherqui D Geller DA . Expert consensus guidelines: how to safely perform minimally invasive anatomic liver resection. J Hepatobiliary Pancreat Sci 2022;29:16–32. 34779150 10.1002/jhbp.1079

[17] Broering D Sturdevant ML Zidan A . Robotic donor hepatectomy: A major breakthrough in living donor liver transplantation. Am J Transplant 2022;22:14–23. 34783439 10.1111/ajt.16889

[18] Ziogas IA Giannis D Esagian SM . Laparoscopic versus robotic major hepatectomy: a systematic review and meta-analysis. Surg Endosc 2021;35:524–535. 32989544 10.1007/s00464-020-08008-2

[19] Zhu L Liu Y Hu M . Comparison of robotic and laparoscopic liver resection in ordinary cases of left lateral sectionectomy. Surg Endosc 2022;36:4923–4931. 34750706 10.1007/s00464-021-08846-8

[20] Hu MG Wang J Yin ZZ . First two-stage robotic ALPPS in HCC patients with hepatic vein invasion: a step-by-step procedure from a clinical case. World J Surg Oncol 2021;19:58. 33612103 10.1186/s12957-021-02170-0 PMC7898755

[21] Zhao ZM Yin ZZ Pan LC . Robotic isolated partial and complete hepatic caudate lobectomy: a single institution experience. Hepatobiliary Pancreat Dis Int 2020;19:435–439. 32513586 10.1016/j.hbpd.2020.05.003

[22] Idrees K Bartlett DL . Robotic liver surgery. Surg Clin North Am 2010;90:761–774. 20637946 10.1016/j.suc.2010.04.020

[23] Lai ECH Tang CN . Training robotic hepatectomy: the Hong Kong experience and perspective. Hepatobiliary Surg Nutr 2017;6:222–229. 28848744 10.21037/hbsn.2017.01.21 PMC5554762

[24] Chong CC Fuks D Lee KF . Propensity score-matched analysis comparing robotic and laparoscopic right and extended right hepatectomy. JAMA Surg 2022;157:436–444. 35262660 10.1001/jamasurg.2022.0161 PMC8908223

[25] Zhao ZM Yin ZZ Pan LC . Robotic anatomic isolated complete caudate lobectomy: left-side approach and techniques. Asian J Surg 2021;44:269–274. 32747143 10.1016/j.asjsur.2020.07.011

[26] Liu Q Zhang T Hu M . Comparison of the learning curves for robotic left and right hemihepatectomy: a prospective cohort study. Int J Surg 2020;81:19–25. 32739547 10.1016/j.ijsu.2020.07.022

[27] Zhao ZM Yin ZZ Meng Y . Successful robotic radical resection of hepatic echinococcosis located in posterosuperior liver segments. World J Gastroenterol 2020;26:2831–2838. 32550758 10.3748/wjg.v26.i21.2831 PMC7284188

[28] Kabir T Syn N Koh YX . Impact of tumor size on the difficulty of minimally invasive liver resection. Eur J Surg Oncol 2022;48:169–176. 34420824 10.1016/j.ejso.2021.08.019

[29] Mathew G Agha R Albrecht J . STROCSS 2021: strengthening the reporting of cohort, cross-sectional and case-control studies in surgery. Int J Surg 2021;96:106165. 34774726 10.1016/j.ijsu.2021.106165

[30] Chen ZL Zhang CW Liang L . Major hepatectomy in elderly patients with large hepatocellular carcinoma: a multicenter retrospective observational study. Cancer Manag Res 2020;12:5607–5618. 32753973 10.2147/CMAR.S258150 PMC7358072

[31] Dumronggittigule W Han HS Komoltri C . Laparoscopic versus open hepatectomy for large hepatocellular carcinoma: a single center propensity-score-matching comparative analysis of perioperative outcomes and long-term survival. Surg Endosc 2023;37:2997–3009. 36520225 10.1007/s00464-022-09812-8

[32] Ruiz E Pineau P Flores C . A preoperative nomogram for predicting long-term survival after resection of large hepatocellular carcinoma (>10 cm). HPB (Oxford) 2022;24:192–201. 34226129 10.1016/j.hpb.2021.06.006

[33] Ikai I Yamamoto Y Yamamoto N . Results of hepatic resection for hepatocellular carcinoma invading major portal and/or hepatic veins. Surg Oncol Clin N Am 2003;12:65–75; ix. 12735130 10.1016/s1055-3207(02)00082-0

[34] Vauthey JN Lauwers GY Esnaola NF . Simplified staging for hepatocellular carcinoma. J Clin Oncol 2002;20:1527–1536. 11896101 10.1200/JCO.2002.20.6.1527

[35] Dindo D Demartines N Clavien PA . Classification of surgical complications: a new proposal with evaluation in a cohort of 6336 patients and results of a survey. Ann Surg 2004;240:205–213. 15273542 10.1097/01.sla.0000133083.54934.ae PMC1360123

[36] Reig M Forner A Rimola J . BCLC strategy for prognosis prediction and treatment recommendation: the 2022 update. J Hepatol 2022;76:681–693. 34801630 10.1016/j.jhep.2021.11.018 PMC8866082

[37] Perri G Marchegiani G Reich F . Intraoperative blood loss estimation in hepato-pancreato-biliary surgery- relevant, not reported, not standardized: results from a systematic review and a worldwide snapshot survey. Ann Surg 2023;277:e849–e855. 35837979 10.1097/SLA.0000000000005536

[38] Zhu P Liao W Ding ZY . Learning curve in robot-assisted laparoscopic liver resection. J Gastrointest Surg 2019;23:1778–1787. 30406576 10.1007/s11605-018-3689-x

[39] Wang ZZ Tang WB Hu MG . Robotic vs laparoscopic hemihepatectomy: a comparative study from a single center. J Surg Oncol 2019;120:646–653. 31313324 10.1002/jso.25640

[40] Hu M Liu Y Li C . Robotic versus laparoscopic liver resection in complex cases of left lateral sectionectomy. Int J Surg 2019;67:54–60. 31121328 10.1016/j.ijsu.2019.05.008

[41] Goh BKP Syn N Koh YX . Comparison between short and long-term outcomes after minimally invasive versus open primary liver resections for hepatocellular carcinoma: a 1:1 matched analysis. J Surg Oncol 2021;124:560–571. 34061361 10.1002/jso.26556

[42] Gavriilidis P Roberts KJ Aldrighetti L . A comparison between robotic, laparoscopic and open hepatectomy: a systematic review and network meta-analysis. Eur J Surg Oncol 2020;46:1214–1224. 32312592 10.1016/j.ejso.2020.03.227

[43] Noh JH Kim TS Ahn KS . Prognostic factors after hepatic resection for the single hepatocellular carcinoma larger than 5 cm. Ann Surg Treat Res 2016;91:104–111. 27617250 10.4174/astr.2016.91.3.104 PMC5016599

[44] Han J Li ZL Xing H . The impact of resection margin and microvascular invasion on long-term prognosis after curative resection of hepatocellular carcinoma: a multi-institutional study. HPB (Oxford) 2019;21:962–971. 30718183 10.1016/j.hpb.2018.11.005

[45] Zhao HC Wu RL Liu FB . A retrospective analysis of long term outcomes in patients undergoing hepatic resection for large (>5 cm) hepatocellular carcinoma. HPB (Oxford) 2016;18:943–949. 27640098 10.1016/j.hpb.2016.08.005 PMC5094476

[46] Wang JC Hou JY Chen JC . Development and validation of prognostic nomograms for single large and huge hepatocellular carcinoma after curative resection. Eur J Cancer 2021;155:85–96. 34371445 10.1016/j.ejca.2021.07.009

[47] European Association for the Study of the Liver. Electronic addresseee, European Association for the Study of the LEASL Clinical Practice Guidelines: Management of hepatocellular carcinoma. J Hepatol 2018;69:182–236. 29628281 10.1016/j.jhep.2018.03.019

[48] Korean Liver Cancer A, National Cancer C . 2018 Korean Liver Cancer association-national cancer center korea practice guidelines for the management of hepatocellular carcinoma. Gut Liver 2019;13:227–299. 31060120 10.5009/gnl19024 PMC6529163

[49] Kudo M Matsui O Izumi N . JSH consensus-based clinical practice guidelines for the management of hepatocellular carcinoma: 2014 update by the liver cancer study group of Japan. Liver Cancer 2014;3:458–468. 26280007 10.1159/000343875 PMC4531423

[50] Ito K Takemura N Inagaki F . Difference in treatment algorithms for hepatocellular carcinoma between world’s principal guidelines. Glob Health Med 2020;2:282–291. 33330822 10.35772/ghm.2020.01066 PMC7731415

[51] Liu Y Wang Y Wei Z . Exploratory study of microparticle transcatheter arterial chemoembolization combined with resection for huge hepatocellular carcinoma. iLIVER 2022;1:35–42. 10.1016/j.iliver.2022.01.001 PMC12212601 40636140

