# Supplementary material for: Short-term and long-term outcomes after robotic versus open hepatectomy in patients with large hepatocellular carcinoma: a multicenter study
Source: Int J Surg. 2023 Nov 16;110(2):660–7. doi: 10.1097/JS9.0000000000000873 (PMC10871596; doi:10.1097/JS9.0000000000000873)
Supplement: Supplementary file 2 [file js9-110-0660-s002.docx]

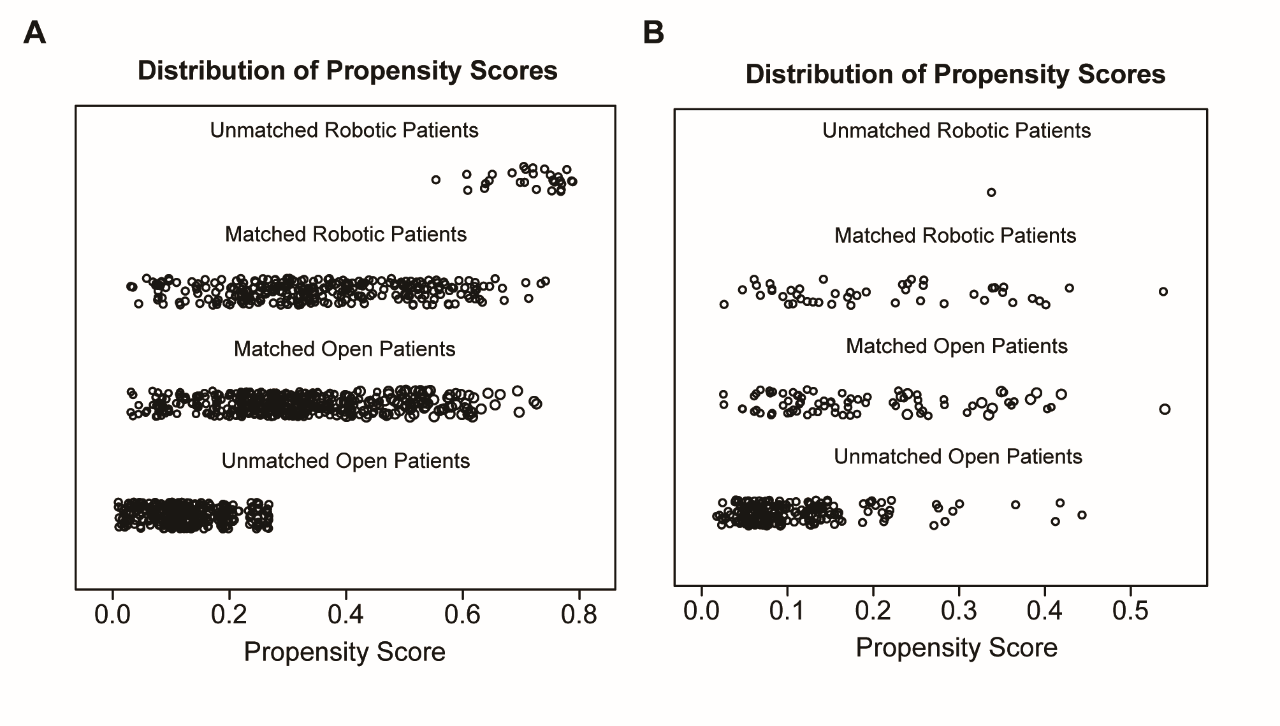
**Supplementary Figure 1.** Standardized mean differences dot plot of PSM. **A.** PSM for all patients; **B.** PSM for huge HCC patients.

**Supplementary Table 1. Survival analyses for all HCC patients after PSM**

| **Time after liver resection** | **Open group** | **Robotic group** | ***P* value** |
| --- | --- | --- | --- |
| 1-year OS rate, (%) | 89.2 | 92.5 |  |
| 3-year OS rate, (%) | 68.9 | 71.9 |  |
| 5-year OS rate, (%) | 53.2 | 55.9 |  |
| Median OS (95% CI), months | 64.4 (56.0-79.0) | 68.9 (55.1-NA) | 0.475 |
| 1-year RFS rate, (%) | 59.4 | 64.7 |  |
| 3-year RFS rate, (%) | 37.1 | 38.8 |  |
| 5-year RFS rate, (%) | 25.6 | 26.0 |  |
| Median RFS (95% CI), months | 20.0 (15.2-23.8) | 25.7 (16.7-31.3) | 0.500 |

**Abbreviation:** HCC, hepatocellular carcinoma, PSM, propensity score matching; OS, overall survival; RFS, recurrence-free survival; CI, confidence interval.

**Supplementary Table 2. Univariable and multivariable cox proportional hazards analyses for RFS**

| **Characteristics** | **Univariable analysis** |  |  | **Multivariable analysis** | |
| --- | --- | --- | --- | --- | --- |
|  | **HR (95% CI)** | ***P* value** |  | **HR (95% CI)** | ***P* value** |
| Age, years, > 60 vs ≤ 60 | 0.894 (0.762-1.048) | 0.167 |  |  |  |
| Sex, male vs female | 0.945 (0.778-1.147) | 0.567 |  |  |  |
| BMI, kg/m^2^ | 0.982 (0.958-1.006) | 0.148 |  |  |  |
| ASA grade, >Ⅱ vs ≤ Ⅱ | 0.889 (0.679-1.164) | 0.392 |  |  |  |
| Viral hepatitis, yes vs no | 1.125 (0.867-1.459) | 0.377 |  |  |  |
| ALB, g/L, < 35 vs ≥ 35 | 0.913 (0.723-1.151) | 0.440 |  |  |  |
| TBIL, mol/L, ≤ 17 vs > 17 | 0.970 (0.820-1.146) | 0.717 |  |  |  |
| AFP, ng/mL, > 400 vs ≤ 400 | 1.438 (1.246-1.660) | <0.001 |  | 1.393 (1.205-1.609) | **<0.001** |
| Lesion size, cm, > 10 vs ≤ 10 | 1.419 (1.216-1.657) | <0.001 |  | 1.381 (1.182-1.613) | **<0.001** |
| No. of tumors, multiple vs solitary | 1.310 (1.042-1.647) | 0.021 |  | 1.258 (1.000-1.582) | 0.050 |
| Cirrhosis, yes vs no | 1.127 (0.968-1.313) | 0.124 |  |  |  |
| Pringle maneuver, yes vs no | 1.150 (0.967-1.367) | 0.114 |  |  |  |
| Total clamping time, min | 1.002 (0.999-1.005) | 0.212 |  |  |  |
| MVI, present vs absent | 1.237 (1.070-1.429) | 0.004 |  | 1.208 (1.045-1.396) | **0.011** |

Bold text hinted that these variables were statistically significant

**Abbreviation:** RFS, recurrence-free survival; HR, Hazard Ratio. CI, confidence interval; ASA, American Society of Anesthesiologists; AFP, α-fetoprotein; ALB, albumin; TBIL, total bilirubin; MVI, Microvascular invasion.

**Supplementary Table 3. Baseline characteristics of** **HCC patients with** **huge tumours in the robotic and open groups before and after PSM**

| **Variable** | **Before PSM** | | |  | **After PSM ¶** | | |
| --- | --- | --- | --- | --- | --- | --- | --- |
|  | **Open group (n=299)** | **Robotic group (n=48)** | ***P* value** |  | **Open group (n=83)** | **Robotic group (n=47)** | ***P* value** |
| Age, years |  |  |  |  |  |  |  |
| ≤ 60 | 240 (80%) | 26 (54%) | <0.001 |  | 49 (59%) | 26 (55%) | 0.680 |
| > 60 | 59 (20%) | 22 (46%) |  |  | 34 (41%) | 21 (45%) |  |
| Sex |  |  |  |  |  |  |  |
| Female | 56 (19%) | 9 (19%) | 0.997 |  | 17 (20%) | 9 (19%) | 0.855 |
| Male | 243 (81%) | 39 (81%) |  |  | 66 (80%) | 38 (81%) |  |
| BMI | 23.66 (22.22-25.73) | 23.83 (22.34-26.52) | 0.873 |  | 23.74 (22.1-25.72) | 23.88 (22.45-26.57) | 0.771 |
| ASA grade |  |  |  |  |  |  |  |
| ≤ Ⅱ | 278 (93%) | 43 (90%) | 0.594 |  | 75 (90%) | 43 (91%) | 1.000 |
| > Ⅱ | 21 (7%) | 5 (10%) |  |  | 8 (10%) | 4 (9%) |  |
| Viral hepatitis |  |  |  |  |  |  |  |
| No | 25 (8%) | 4 (8%) | 1.000 |  | 5 (6%) | 4 (9%) | 0.859 |
| Yes | 274 (92%) | 44 (92%) |  |  | 78 (94%) | 43 (91%) |  |
| Cirrhosis |  |  |  |  |  |  |  |
| No | 112 (37%) | 16 (33%) | 0.582 |  | 29 (35%) | 15 (32%) | 0.726 |
| Yes | 187 (63%) | 32 (67%) |  |  | 54 (65%) | 32 (68%) |  |
| ALB, g/L |  |  |  |  |  |  |  |
| < 35 | 41 (14%) | 5 (10%) | 0.532 |  | 6 (7%) | 5 (11%) | 0.732 |
| ≥ 35 | 258 (86%) | 43 (90%) |  |  | 77 (93%) | 42 (89%) |  |
| TBIL, mol/L |  |  |  |  |  |  |  |
| ≤ 17 | 224 (75%) | 38 (79%) | 0.525 |  | 64 (77%) | 37 (79%) | 0.832 |
| > 17 | 75 (25%) | 10 (21%) |  |  | 19 (23%) | 10 (21%) |  |
| INR | 1.07 (1.00-1.16) | 1.07 (1.02-1.12) | 0.840 |  | 1.05 (0.99-1.15) | 1.07 (1.02-1.12) | 0.280 |
| AFP, ng/mL |  |  |  |  |  |  |  |
| ≤ 400 | 144 (48%) | 25 (52%) | 0.614 |  | 42 (51%) | 24 (51%) | 0.960 |
| > 400 | 155 (52%) | 23 (48%) |  |  | 41 (49%) | 23 (49%) |  |
| Varices |  |  |  |  |  |  |  |
| No | 275 (92%) | 46 (96%) | 0.517 |  | 76 (92%) | 45 (96%) | 0.588 |
| Yes | 24 (8%) | 2 (4%) |  |  | 7 (8%) | 2 (4%) |  |
| No. of tumors |  |  |  |  |  |  |  |
| Solitary | 270 (90%) | 39 (81%) | 0.062 |  | 69 (83%) | 38 (81%) | 0.743 |
| Multiple | 29 (10%) | 9 (19%) |  |  | 14 (17%) | 9 (19%) |  |
| MVI |  |  |  |  |  |  |  |
| Absent | 130 (43%) | 28 (58%) | 0.055 |  | 47 (57%) | 27 (57%) | 0.928 |
| Present | 169 (57%) | 20 (42%) |  |  | 36 (43%) | 20 (43%) |  |

Data are presented as n (%) or median (interquartile range). ¶, because some cases could not simultaneously find effective matching objects, the matching result was not an absolute 1:2.

**Abbreviation:** HCC, hepatocellular carcinoma, PSM, propensity score matching; ASA, American Society of Anesthesiologists; AFP, α-fetoprotein; ALB, albumin; TBIL, total bilirubin; INR, international normalized ratio; MVI, microvascular invasion.

**Supplementary Table 4. Survival analyses for HCC patients with huge tumor after PSM**

| **Time after liver resection** | **Open group** | **Robotic group** | ***P* value** |
| --- | --- | --- | --- |
| 1-year OS rate, (%) | 82.5 | 83.4 |  |
| 3-year OS rate, (%) | 52.2 | 61.6 |  |
| 5-year OS rate, (%) | 40.8 | 35.2 |  |
| Median OS (95% CI), months | 43.3 (25.9-86.9) | 44.8 (32.3-NA) | 0.894 |
| 1-year RFS rate, (%) | 49.3 | 53.4 |  |
| 3-year RFS rate, (%) | 29.7 | 32.9 |  |
| 5-year RFS rate, (%) | 21.6 | 24.7 |  |
| Median RFS (95% CI), months | 9.8 (6.0-20.0) | 20.0 (7.8-39.2) | 0.450 |

**Abbreviation:** HCC, hepatocellular carcinoma, PSM, propensity score matching; OS, overall survival; RFS, recurrence-free survival; CI, confidence interval.
